# Antifungal Peptides SmAP_α1–21_ and SmAP_γ27–44_ Designed from Different Loops of DefSm2-D Have Distinct Modes of Action

**DOI:** 10.3390/antibiotics14050430

**Published:** 2025-04-24

**Authors:** Micaela Iturralde, Juan Pablo Bracho, Jessica A. Valdivia-Pérez, Fanny Guzmán, Ismael Malbrán, Sabina María Maté, María Laura Fanani, Sandra Vairo Cavalli

**Affiliations:** 1 Centro de Investigación de Proteínas Vegetales (CIPROVE), Departamento de Ciencias Biológicas, Facultad de Ciencias Exactas, Universidad Nacional de La Plata (UNLP), La Plata 1900, Argentina; micaelaiturralde@biol.unlp.edu.ar (M.I.); juanpablobracho@biol.unlp.edu.ar (J.P.B.); 2 Departamento de Química Biológica Ranwel Caputto, Facultad de Ciencias Químicas, Universidad Nacional de Córdoba, Córdoba 5000, Argentina; jessica.perez@unc.edu.ar; 3 Centro de Investigaciones en Química Biológica de Córdoba (CIQUIBIC), Consejo Nacional de Investigaciones Científicas y Técnicas (CONICET), Córdoba 5000, Argentina; 4 Núcleo de Biotecnología Curauma (NBC), Pontificia Universidad Católica de Valparaíso, Valparaíso 2373223, Chile; fanny.guzman@pucv.cl; 5 Centro de Investigaciones de Fitopatología (CIDEFI-UNLP-CIC), Facultad de Ciencias Agrarias y Forestales, Universidad Nacional de La Plata (UNLP), La Plata 1900, Argentina; ismael.malbran@agro.unlp.edu.ar; 6 INIBIOLP, CCT-La Plata, CONICET, Facultad de Ciencias Médicas, Universidad Nacional de La Plata (UNLP), La Plata 1900, Argentina; smate@med.unlp.edu.ar

**Keywords:** antifungal peptides, defensin-derived peptides, *Silybum marianum*, *Fusarium graminearum*, peptide–membrane interaction

## Abstract

**Background:** The use of antimicrobial peptides (AMPs) as biotechnological tools is an area of growing interest in the research that seeks to improve crop defense. SmAP_α1–21_ and SmAP_γ27–44_ were previously reported to inhibit *Fusarium graminearum*, permeabilize the plasma membrane and induce cytoplasmic disorganization. To exert its activity, SmAP_α1–21_ initially enters through the basal and apical cells of *F. graminearum* conidia and then displays a general but non-homogeneous distribution in the cytoplasm of all conidial cells, in contrast. **Methods:** We analyzed, focusing on membrane interaction, the mode of action of SmAP_γ27–44_, a peptide based on the γ-core of defensins DefSm2-D and DefSm3, and SmAP_α1–21_, based on the α-core of DefSm2-D. Additionally, we compared the behavior of SmAP_α1–21_ with that of SmAP3_α1–21_ based on DefSm3 but with no activity against *F. graminearum*. **Results:** In this study, we showed that SmAP_γ27–44_ enters the cells with discrete intracellular localization. Furthermore, both peptides disrupted the plasma membrane, but with different modes of action. When large unilamellar liposomes (LUVs) containing phosphatidic acid and ergosterol were used as a filamentous fungal plasma membrane model, SmAP_γ27–44_ strongly induced aggregation concomitantly with the solubilization of the liposomes and showed the maximal insertion of its tryptophan moiety into the membrane’s hydrophobic interior. In comparison, SmAP_α1–21_ showed a high effect on the ζ potential of anionic vesicles, vesicle aggregation capacity after reaching a concentration threshold, and moderate transfer of tryptophan to the membrane. SmAP3_α1–21_, on the other hand, showed poor superficial adsorption to liposomes. **Conclusions:** In view of our results, a cell penetration peptide-like effect was pictured for the γ-core defensin-derived peptide and a classical AMP action was observed for the α-core defensin-derived one.

## 1. Introduction

Modern intensive agriculture relies heavily on poorly degradable chemical fungicides to manage diseases caused by fungi and oomycetes in the field and the harvested products. The accumulation of fungicides in soil, plants, and water can cause significant environmental toxicity and human and animal health problems [1]. Hence, developing non-toxic eco-innovative alternatives to manage fungal plant infections has become a top priority [2,3]. One environmentally friendly strategy includes using plant-derived antimicrobials as biological control agents [4]. The different modes of action of antimicrobial plant proteins and peptides, which are effector molecules of innate immunity, make them promising alternatives to the current antifungal agents. In this regard, antimicrobial peptides (AMPs), particularly those with multisite target modes of action, have been proposed as therapeutic molecules due to their low toxicity for plant and mammalian cells [5,6,7]. Plant antifungal peptides are generally considered safe for human cells, as they act on specific microbial components [8].

An AMP’s antimicrobial activity is related to its primary structure and chemical and physicochemical properties, namely the presence and relative quantity of positively charged residues, the hydrophobicity, the net charge, and the helicity of the structure [9,10]. Furthermore, the rational design from plant AMPs provides a sound basis for developing new molecules with enhanced features [11].

Plant defensins belong to a large family of cysteine-rich cationic AMPs with diverse mechanisms of action and functions; most of them exhibit antifungal activity against a wide range of fungi. Plant genomes contain multiple defensin genes. This gene prolixity is accepted to be the result of the coevolution of the plant’s defensive systems and the pathogen, and it is necessary to protect the plant against pathogens with greater tolerance to one type of defense molecule [12,13].

In terms of the structure, all defensins share a common cysteine-stabilized three-dimensional fold. Differences in defensin activity, specificity, potency, or mode of action are likely due to variations in the primary structure and the charge distribution of solvent-exposed loops. One of the loops is part of the γ-core motif GXC(X_3–9_)C, a β-hairpin structure that is not exclusive to the defensin subclass but is conserved across all cysteine-rich AMPs. For example, the replacement of two residues (E11 and H12) from the γ-core sequence of the AMP PeAfpB of *Penicillium expansum* by those corresponding to the AMP PeAfpA (K10 and D11) increased the antifungal activity of the chimera protein and made it similar in specificity to PeAfpA [11]. In addition to a γ-core motif, plant defensins feature an α-core motif characterized by the consensus sequence GXC(X_3–5_)C. However, this motif is not conserved across all disulfide-stabilized AMPs. It is located in the loop between the β1 strand and the α-helix, partially overlapping the latter. Unlike the γ-core motif, the α-core lacks the β hairpin structure, and its role in activity has been less thoroughly investigated [14].

Even when the mechanism of action of a small number of plant defensins has been clarified, these mechanisms are complex and not fully understood [15,16,17]. For example, defensin Ppdef1 (from *Picramnia pentandra*) inhibits the growth of the dermatophytic fungus *Trichophyton rubrum*; Ppdef1 induces fungal cells’ mitochondria to produce reactive oxygen species (ROS) that destabilize the plasma membrane [15]. MtDef4 (from *Medicago trucatula*) inhibits the plant pathogen *Botrytis cinerea* by first binding cell wall components and cell membrane phospholipids, then penetrating the plasma membrane, and finally entering the cell, where it specifically binds to ribosomes to inhibit protein translation [7].

Short peptide fragments corresponding to the functional sequences responsible for an AMP antimicrobial activity, such as the γ-core motif [18], can be used as templates to create new antimicrobial agents for agriculture and medicine [4,17,19]. There are examples of peptides spanning the γ-core and extending toward the C-terminus that display inhibitory activity against species of the genus Fusarium in the literature. Sathoff et al., 2019 [20], designed peptides containing the γ-core motif and extended six residues into the C-terminal region of parent defensins MsDef1 (*M. sativa*), MtDef4 and MtDef5 (*M. truncatula*), RsAFP-2 (*Raphanus sativa*), and So-D2 (*Spinacia oleracea*). These peptides show activity against plant and animal pathogens, and those derived from MtDef4 and MtDef5 inhibited the growth of *F. solani*. While not all these peptides were effective against every pathogen tested by the authors, those that showed activity did so within the micromolar range. In a study conducted by Slezina et al. [19], antimicrobial peptides were also designed to span the γ-core toward the C-terminus of various cysteine-rich AMPs, including defensins, snakins, non-specific lipid-transfer proteins, and thionin- and knottin-like peptides. Among these peptides, the most active against species of the genus Fusarium were the 18-amino acid peptides derived from defensins, DELF1-16_65–82_ and DELF1-36_65–82_, which exhibited activity in the micromolar range against *F. culmorum* and *F. oxysporum*. To ensure their applicability, a thorough understanding of the structure and function of AMPs is crucial, because it enables protein engineering and the rational design of enhanced AMPs [14].

In previous works [14,21], our group designed and synthesized the peptides SmAP_γ27–44_ and SmAP_α1–21_ from the γ- and α-core motifs of defensin DefSm2-D from the wild thistle *Silybum marianum*, which is an important medicinal and edible plant used since ancient times [22,23]. Both peptides were shown to inhibit *F. graminearum* with a multistep mechanism of action that includes membrane permeabilization [14]. Here, we continued the study of the modes of action of both peptides, specifically their interaction with model membrane systems. Additionally, we designed the peptide SmAP3_α1–21_ based on the α-core motif of DefSm3 (*S. marianum*); this defensin shares its γ-core with DefSm2-D, and the peptide SmAP_γ27–44_ has a common sequence in both defensins.

## 2. Results

### 2.1. Peptides Characterization

**Figure 1:**
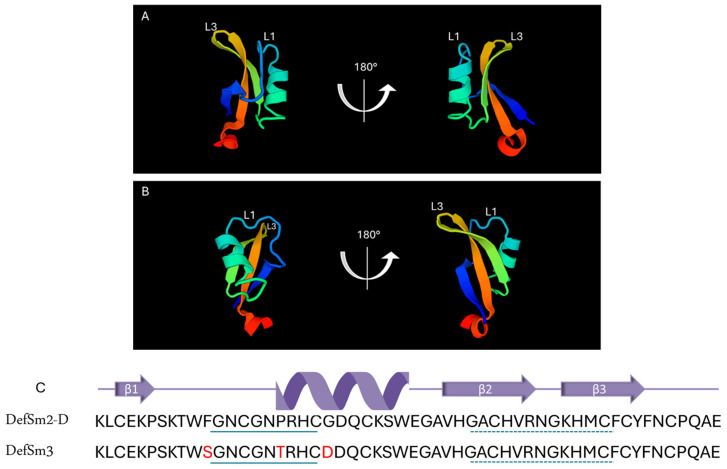
Structural characterization of defensins DefSm2-D and DefSm3. Two views of DefSm2-D (**A**) and DefSm3 (**B**) structures in cartoon representation. L1: α-core, L3: γ-core. (**C**) Amino acid sequence and secondary structure prediction of DefSm2-D and DefSm3. In both defensins, the α-core motif is underlined with a continuous line, while the γ-core is underlined with a dashed line.

The BLAST^®^ protein (blastp suite, https://blast.ncbi.nlm.nih.gov/Blast.cgi, accessed on 1 October 2024) analysis of the DefSm3-deduced amino acid sequence (accession number PP895202) revealed a 94% identity with DefSm2-D, 90% identity with DmAMP1 from seeds of *Dahlia merckii* [24] (accession number P0C8Y4, http://www.camp4.bicnirrh.res.in/seqDisp.php?id=CAMPSQ1167, accessed on 10 October 2024), and 84% identity with Ha-DEF1 from roots of *Helianthus annuus* [25] (http://www.camp4.bicnirrh.res.in/seqDisp.php?id=CAMPSQ3297, accessed on 10 October 2024). Changes between DefSm3 and DefSm2-D are located from positions 11 to 21 ([F11S], [P17T], [G21D]). Figure 1 shows the highly conserved structures of DefSm2D and DefSm3 obtained using the structure prediction by AlphaFold. Both models present the stable structural scaffold CSαβ. The global Root Mean Square Deviation (RMSD) comparison between both defensin models revealed a value near 0.5 Å. RMSD quantifies the average distance between atoms of structurally aligned proteins [26]. The smaller the RMSD value, the closer the models are to each other. This implies that the three amino acid changes do not cause variations in the global structure.

**Figure 2:**
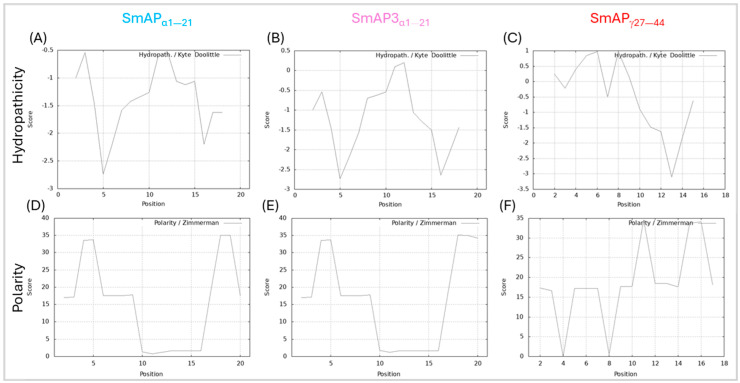
Hydropathy and polarity profiles. Hydropathy in the top row, according to the Kyte–Doolittle algorithm (scale from −3.5 to 1, on the ordinate axis and position of the amino acids on the abscissa axis). Polarity according to Zimmerman’s algorithm (scale from 0 to 40, on the ordinate axis and position of the amino acids on the abscissa axis). (**A**,**D**) SmAP_α1–21_, (**B**,**E**) SmAP3_α1–21_, and (**C**,**F**) SmAP_γ27–44_.

**Figure 3:**
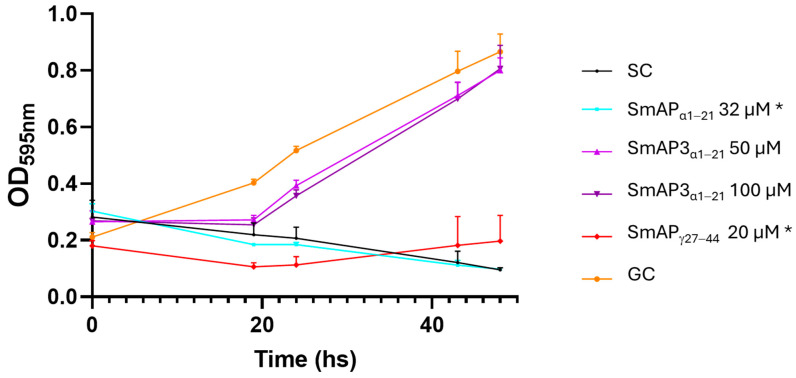
Growth curves of *F. graminearum* in the presence of different concentrations of DefSm2-D- and DefSm3-derived peptides. Error bars represent the standard deviation of technical triplicates and two independent assays (n = 6). One-way ANOVA was carried out. Asterisks indicate significant differences with the growth control; (*) *p* < 0.05. OD_595 nm_ is the optical density at 595 nm. SC: Sterility control. GC: Growth control.

SmAP3_α1–21_ has a lower isoelectric point (pI), net charge, and grand average of hydropathy (GRAVY) than SmAP_α1–21_ due to the three amino acid residue changes. Table 1 summarizes the main features of the three peptides studied. According to the results shown in Figure 2, the hydropathy and polarity profiles of SmAP_α1–21_ and SmAP3_α1–21_ are similar, but SmAP_α1–21_ is more hydrophobic than SmAP3_α1–21_ as indicated by its GRAVY value. As shown in Figure 3, SmAP3_α1–21_ does not inhibit *F. graminearum* fungal growth at 50 or 100 μM. However, peptides derived from DefSm2-D show 100% inhibitory activity against *F. graminearum* at the previously reported minimum inhibitory concentration (MIC) [13].

### 2.2. Peptides—Vesicle Interaction Induces Membrane Remodeling

A lipid mixture composed of phosphatidylcholine (PC)/phosphatidylethanolamine (PE)/phosphatidic acid (PA)/ergosterol (Erg) was designed to imitate the plasma membrane of filamentous fungi, such as *F. graminearum*. Figure S2 (Supplementary Materials) shows that the mixed monolayer behaves as a liquid-expanded phase with a compressibility modulus of 101 ± 9 mN/m at a surface pressure of 30 mN/m. The mixed monolayer also showed a collapse pressure of 45 ± 2 mN/m. The isotherm curves of pure components were checked to agree with the bibliography: POPC [27], POPE [28], ergosterol [29], and DLPA [30].

The designed lipid mixture successfully formed large unilamellar vesicles (LUVs) of ~100 nm diameter and ζ potential of −67 ± 8 mV. This parameter is the potential at the slipping/shear plane of a colloid particle moving under an electric field and depends on the net charge of the particle surface as well as on the ion double layer that is formed at the surface vicinity [31]. Therefore, the ζ potential of the vesicles should be affected by the surface union of cationic molecules, such as the defensin-derived peptides [32].

**Figure 4:**
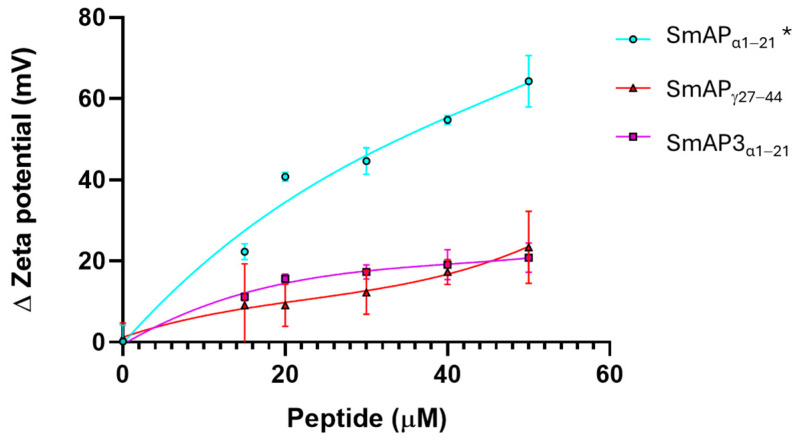
ζ potential neutralization by the interaction with defensin-derived peptides. ζ potential increased upon the addition of SmAP_γ27–44_ (red), SmAP_α1–21_ (cyan), or SmAP3_α1–21_ (violet symbols). The ζ potential of large unilamellar vesicles (LUVs) in the absence of peptides was −67 ± 8 mV. Data represent the average of quintuplicates and two independent experiments (n = 10), and error bars denote the standard deviation. One-way ANOVA was carried out. (*) indicates significant differences in the multiple comparisons mode. ζ potential of LUVs challenged with SmAP_α1–21_ presents significant differences compared to the ζ potential of LUVs in the presence of SmAP_γ27–44_ and SmAP3_α1–21_, while these values do not differ between them.

Figure 4 shows that the normalized values of the ζ potential increase with peptide addition. SmAP_α1–21_ shows a significant increase in the ζ potential with peptide addition, which does not reach a maximum, even at concentrations twofold its MIC value. This curve can be modeled by a rectangular hyperbola characterized by an asymptote, which describes a maximum possible ζ potential at 69.6 mV, and 66.3 μM was the peptide concentration at which half this value was reached (C_50_). The two remnant peptides showed a poor increase in the ζ potential in the 0−50 μM concentration range. SmAP3_α1–21_ reached a maximum value of −32.7 mV and a C_50_ of 20 μM. On the contrary, SmAP_γ27–44_ showed a sigmoidal curve, and the maximum ζ potential at a high peptide concentration could not be calculated.

**Figure 5:**
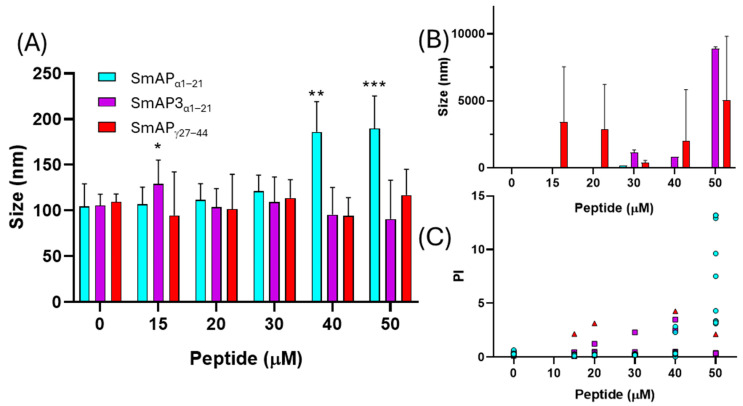
Vesicle aggregation upon peptide addition. LUVs after incubation with different peptide concentrations. (**A**) Data show the first size peak mean with standard deviation (columns). (**B**) Second size peaks of particle diameter. (**C**) Polydispersity index of the particle diameter histograms. Data correspond to the average of ten measurements acquired in two independent experiments. Peptides are: SmAP_γ27–44_ (red triangles), SmAP_α1–21_ (cyan circles), or SmAP3_α1–21_ (violet squares). Two-way ANOVA was carried out. Asterisks indicate significant differences at each peptide concentration between the different peptides, (*): *p* < 0.05; (**): *p* < 0.0001; (***): *p* < 0.0001.

Figure 5 shows the changes in particle size observed upon the incubation of LUVs with the peptides. SmAP_α1–21_, which underwent a partial neutralization of the ζ potential above its MIC (32 μM), showed clear signs of vesicle aggregation in these conditions, reflected in an increase in the particle diameter peak and an increase in the polydispersity index of the size population (Figure 5A,C). On the other hand, SmAP_γ27–44_, the most active peptide against *F. graminearum* (MIC 20 μM), induced vesicle size destabilization, resulting in both smaller and larger particles than control particles at concentrations as low as 15 μM (see the occurrence of a second micrometric size peak in Figure 5B). Finally, SmAP3_α1–21_, a peptide designed from the α-core of DefSm3 with no activity against *F. graminearum*, did not show the neutralization of LUVs (Figure 4) and showed poor vesicle-size-remodeling activity up to 50 μM (Figure 5).

### 2.3. Peptides—Model Membrane Interaction Assessed by Tryptophan Fluorescence

**Figure 6:**
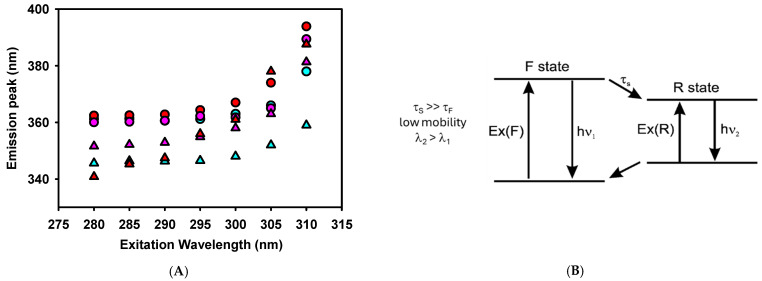
(**A**). Red edge excitation shift (REES) spectroscopy of tryptophan for the defensin-derived peptides in the absence (circles) and presence (triangles) of 1 mM LUVs. Peptides are: SmAP_γ27–44_ (red), SmAP_α1–21_ (cyan), or SmAP3_α1–21_ (violet) at 50 μM concentration. (**B**). Fluorescence behavior when solvent molecules around W have lower mobility (τ_S_) than the fluorophore (W, τ_F_). Modified from Ref. [34].

The spectroscopic changes in tryptophan (W) residues were studied to characterize the peptide–model membrane interaction. Figure 6 shows that all the defensin-derived peptides studied had an emission peak at ~360 nm, comparable with other peptides that predominantly had a random-coil secondary structure in an aqueous solution [33]. In the presence of LUVs, the peptides showed a blue shift of the W fluorescence emission (Figure 6A and Table 2) that is inversely proportional to their MIC.

We further studied W fluorescence modulation by peptides interacting with the model lipid membrane through red edge excitation shift (REES) spectroscopy. This technique provides information about the relaxation of the solvent environment of the W residue [33,34]. This phenomenon arises from the observation that the emission spectra of polar fluorophores, such as W, can shift to longer wavelengths as the excitation wavelength moves further toward the red end (longer wavelength) of the probe’s absorption spectrum in moderately polar and viscous solvents.

A two-state model presents a conceptual framework to explain the observed effects of REES (Figure 6B). With a wavelength central to the absorption band, fluorophores are excited to an initially excited state (F), around which solvent reorientation did not occur. This excited state can relax to a lower energy state (R) through a process characterized by a specific lifetime that involves solvent reorientation. The relaxation rate depends on the interactions between the fluorophore and the surrounding solvent molecules and the speed at which these interactions adjust to the altered dipole moment of the new excited state [35]. When the solvent reorientation time (τ_S_) is significantly shorter than the fluorescence lifetime of the fluorophore (τ_F_), the solvent molecules can fully reorient before the fluorophore emits. In this scenario, the relaxed emission is unaffected by the excitation wavelength (no REES is observed). This occurs in peptides where the W residue is either fully exposed to a rapidly relaxing solvent or located within a hydrophobic region of the peptide, where the presence of nearby polar groups is minimal [34]. However, this was not the case for the defensin-based peptides studied here, as shown in Figure 6A and Table 2.

In a viscous environment, τ_S_, which depends on the rate of the physical reorientation of solvent molecules, becomes significantly longer than τ_F_. Then, the blue-shifted emission of the F state is observed with normal excitation energy and the emission becomes excitation-dependent. However, when the system is excited with lower energy, a subset of the fluorophores surrounded by the oriented solvent dipoles is selectively excited. This reduces the energy difference between the ground and excited states, leading to the photoselection of fluorophores that interact most strongly with the polar solvent molecules. This effect is expected when the W residue of a peptide remains in an environment with restrictive dipolar interactions [34].

Figure 6A shows a red shift of the W fluorescence emission peak when the excitation wavelength is shifted from 280 to 310 nm. SmAP_γ27–44_, the more active peptide against *F. graminearum*, showed a notable REES in the absence of vesicles compared to other reported membrane-interacting peptides [33,36]. This denotes a strong polar interaction of W with the rest of the peptide residues in an aqueous solution. W is located at the N-terminus of SmAP_γ27–44_, suggesting that this chain extreme may fold to interact with a possible cage-like structure. In the presence of LUVs, SmAP_γ27–44_ showed even higher values of REES, denoting a further degree of structuring induced by its insertion in a viscous environment, such as a lipid membrane.

On the other hand, SmAP_α1–21_ reduces its REES values when transitioning from an aqueous solution to the membrane environment. Considering that SmAP_α1–21_ showed important changes in the ζ potential of LUVs, the reduction in REES may indicate a change in the W environment from a close interaction with polar amino acids of the peptide to a surface position at the vesicle–water interface. In the latter situation, W would sense a loosened interaction with the lipid head groups rather than the polar residues of the peptide. Finally, SmAP3_α1–21_, which did not exhibit antifungal activity against *F. graminearum*, showed high values of REES both in the presence and absence of LUVs. Considering that this peptide shows a scarce blue shift at a 280 nm excitation wavelength, the results suggest that the W residue is in the interior of the peptide strongly interacting with their polar amino acids and does not show significant structural changes upon a weak interaction with the lipid membrane.

It is important to recall that both previously studied peptides (SmAP_γ27–44_ and SmAP_α1–21_) showed no evidence of a secondary structure by circular dichroism spectroscopy in solution [14]. However, W fluorescence can sense a strong dipolar interaction with its neighbor amino acid, which does not require adopting a defined secondary structure for the peptides.

### 2.4. Interaction of Peptides with F. graminearum Macroconidia

**Figure 7:**
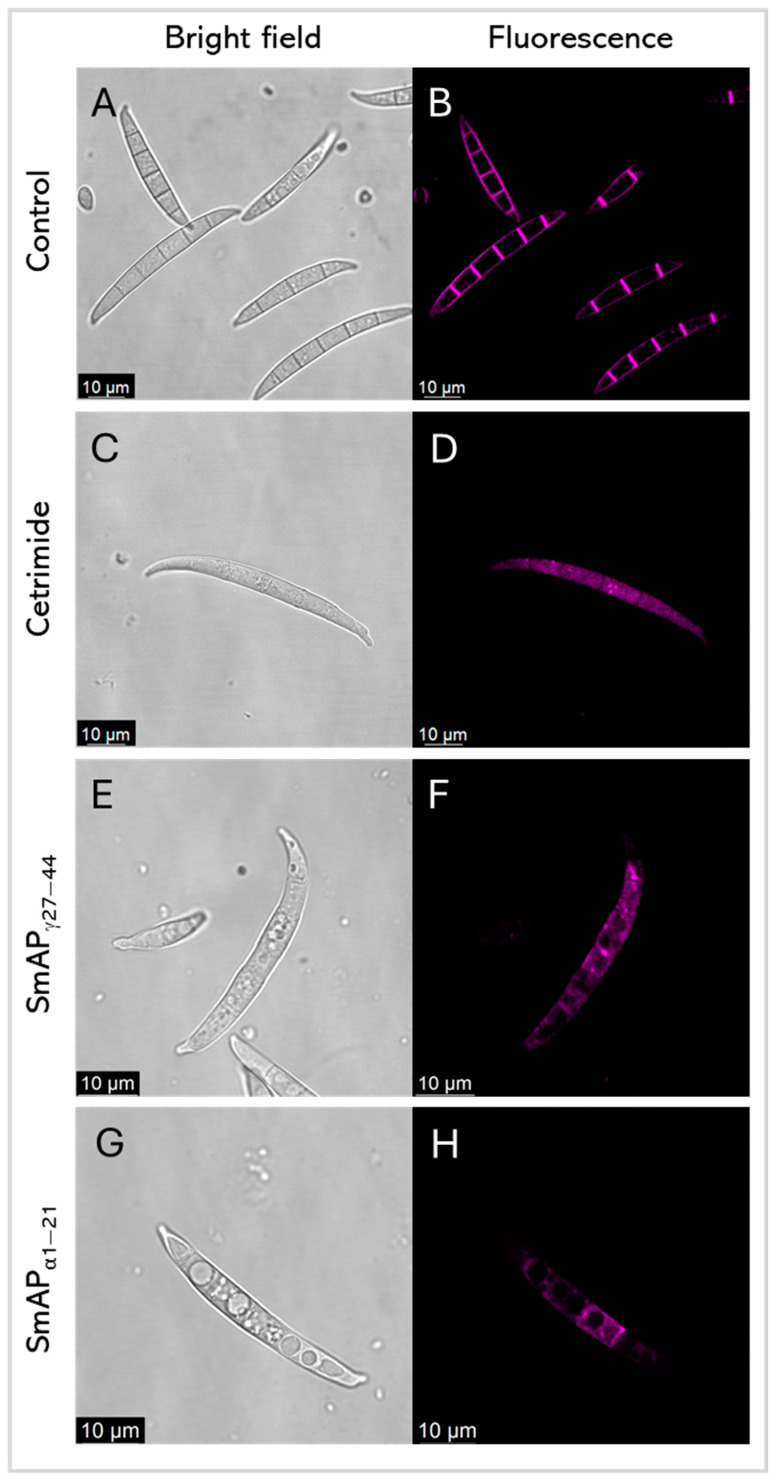
Representative confocal fluorescence images show FM4–64 dye intake by *F. graminearum* in response to cetrimide (**C**,**D**), SmAP_γ27–44_ treatment (**E**,**F**), and SmAP_α1–21_ treatment (**G**,**H**). (**A**,**B**) show conidia with water as the control. Bright-field and fluorescence images are shown as different columns.

Figure 7 shows FM 4–64 intake by *F. graminearum* in response to the SmAP_γ27–44_ or SmAP_α1–21_ challenge, by confocal laser scanning microscopy (CLSM). FM 4–64 is a small amphiphilic molecule that reversibly associates with the outer leaflet of the lipid bilayer of biological membranes but cannot penetrate the cell membrane. It is non-fluorescent in an aqueous solution, but exhibits intense fluorescence upon incorporation into the plasma membrane, as can be seen in conidia treated with water as the negative control (Figure 7B). This dye is useful for tracking plasma membranes, endocytosis/exocytosis, and organelle dynamics [37]. After the incubation of conidia with each peptide, samples were exposed to FM 4–64. Fluorescence was not observed in the plasma membrane of treated conidia, as is the case with the negative control; nevertheless, the intracellular influx of FM 4–64 after the challenge with both peptides and the detergent cetrimide, as a control, may suggest pronounced membrane damage. Also, peptide treatments produce intense vesiculation and different fluorescence profiles. This can indicate different distributions of lipophilic molecules, or hydrophilic zones where the FM 4–64 dye shows low affinity.

**Figure 8:**
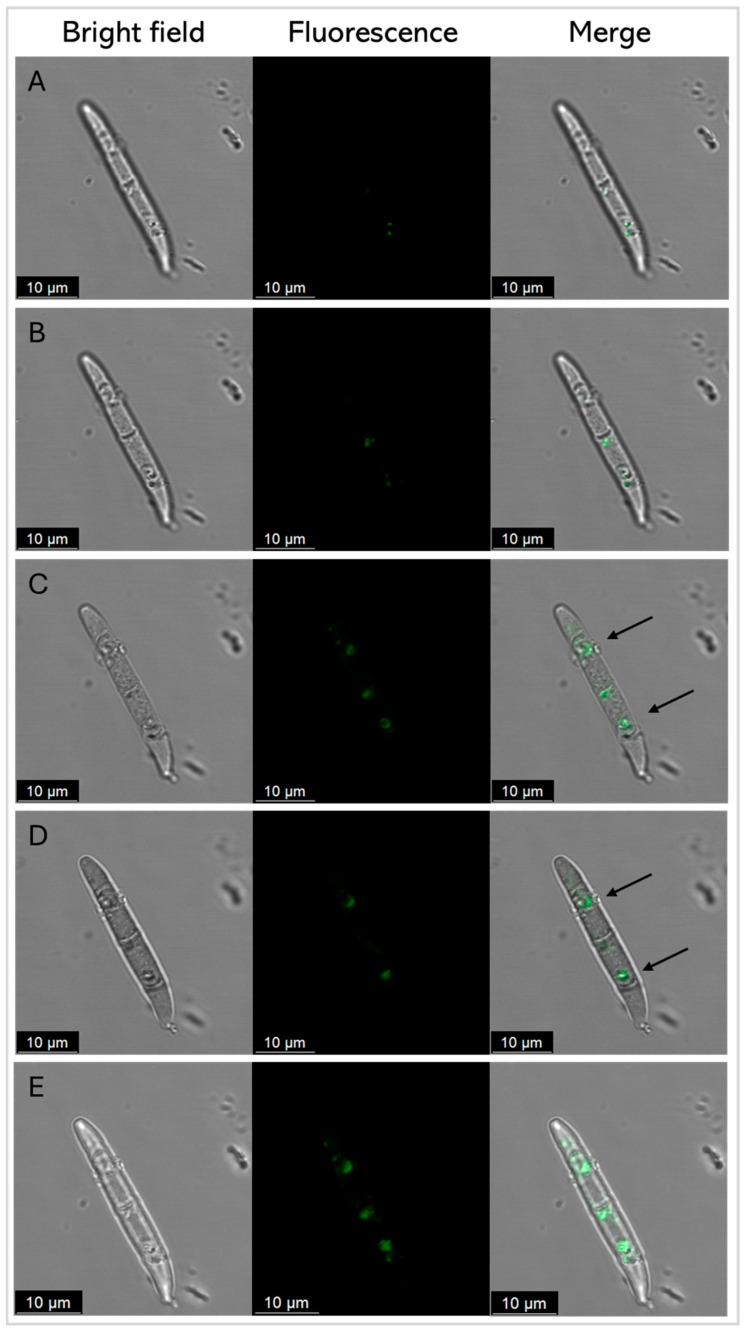
Representative confocal fluorescence images of *F. graminearum* conidia treated with SmAP_γ27–44_ labeled with fluorescein, at its MIC (20 µM) after 45 min. Z total deep = 5.2 µm. (**A**–**D**) Each row represents a 1.3 μm-thick slice of the conidium. Row (**E**) shows the Z-stack maximum-intensity projection. Bright-field, fluorescence, and merger images are shown as different columns. For clarity, arrows show the discrete localization of the labeled peptide in the merger.

The fluorescently labeled SmAP_γ27–44_ uptake by conidia cells was further analyzed by CLSM to examine the correlation between antifungal activity and peptide localization Figure 8 shows several images of representative conidia in different planes on the *Z*-axis (A-D), and row E shows the Z-stack maximum-intensity projection. At the incubation time, green fluorescence was found in discrete localizations in each conidium cell.

## 3. Discussion

From a cDNA library synthesized from the total RNA of wild thistle *S. marianum* flowers with primers designed from the conserved N-terminal ends of Asteraceae defensins, our group previously designed antifungal peptides based on the DefSm2-D sequence: SmAP_γ27–44_ and SmAP_α1–21_ from the γ- and α-core plant defensin motifs, respectively. In this study, we deepened the analysis of the mechanisms of action of these two peptides focusing on membrane interaction and compared the behavior of SmAP_α1–21_ with that of SmAP3_α1–21_.

SmAP3_α1–21_ is based on the sequence of DefSm3 from the same cDNA library, with only three differential amino acid residues. Pairs of highly similar sequences of defensins in plant genomes have been proposed as recent duplications with relevance in defensin evolution [38]. Using structure prediction, we showed that the change in the primary structure does not produce notable changes in the tertiary structure of both defensins. On the other hand, changes in specific residues can vary the specificity or the functionality of a defensin; moreover, marginal changes in sequence are usually accompanied by significant changes in antimicrobial activity [17,35]. RsAFP1 and RsAFP2 are nearly identical defensins from radish seeds (*Raphanus sativus*), with two variations at positions [E5Q] and [N27R] in RsAFP1 and RsAFP2, respectively. These substitutions made RsAFP2 one-to-two orders of magnitude more potent against the fungi *Colletotrichum lindemuthianum* and *F. oxysporum* than RsAFP1, while the latter is more active than RsAFP2 against the oomycete *Phytophthora infestans* [39]. Amino acid residues in positions 11, 17, and 21 of DefSm3 are conserved in Ha-DEF1, a sunflower defensin that shares 84% identity with DefSm3 and induces cell death in the parasitic weed broomrape (*Orobanche* sp.) [25].

The γ-core is considered the major determinant of the antifungal activity of defensins. However, peptides derived from the α-core motif and its adjacent regions also inhibit fungal growth at micromolar concentrations. SmAP_α1–21_ and SmAP_γ27–44_ have fungicide activity against *F. graminearum* at low micromolar concentrations [14]. Extension with some residues toward the defensin N- or C-terminal sequence improves α- and γ-core-derived peptides activity, probably due to the inclusion of positively charged or hydrophobic residues not necessarily present in the motif itself [14].

Many antifungal AMPs possess mechanisms of action that involve interacting with the surfaces of fungal cells and with intracellular targets [40]. Defensin OefDef1.1 from *Olea europaea* binds to the cell walls of the plant pathogen *Botrytis cinerea* conidia and germlings, disrupting the plasma membrane; membrane permeabilization appears to be the major factor contributing to OefDef1.1 antifungal activity [41]. GMA4CG_V6, a 17-amino acid peptide containing the γ-core motif of MtDef4, exhibits antifungal activity against *B. cinerea* in vitro. When sprayed on *Nicotiana benthamiana*, rose petals, and tomato fruits, it shows preventative and curative antifungal effects. GMA4CG_V6 localizes in the plasma membrane, subsequently causes membrane permeabilization, and is finally internalized into the vacuole and cytoplasm [17]. SmAP_α1–21_ and SmAP_γ27–44_ also permeabilize the membrane and cause the disorganization of the cytoplasm ultrastructure of treated *F. graminearum* cells. The mechanism of action of SmAP_α1–21_ has specific features that differ from those of SmAP_γ27–44_. For instance, SmAP_α1–21_ induces fungal cell wall alteration and peroxisome biogenesis [21]. Additionally, as demonstrated in the present work, SmAP_γ27–44_ is found in discrete localizations of the fungal cells at the time to kill. In contrast, SmAP_α1–21_ initially enters through the basal and apical cells of *F. graminearum* conidia and, at the time–kill, exhibits a non-uniform distribution throughout the cytoplasm of all conidial cells, while SmAP2H19R and SmAP2H19A, two peptides based on SmAP_α1–21_, localize toward the extracellular region of the conidia to exert their antifungal activity [21].

SmAP_γ27–44_, SmAP_α1–21_, and SmAP3_α1–21_ have an amphipathic nature. Amphiphilicity enables them to be soluble in an aqueous environment and to interact with lipid membranes. For amphipathic AMPs, electrostatic and hydrophobic interactions are critical for activity [25,42]. Fernández et al. [21] previously noticed that the net positive charge of SmAP_α1–21_ is important for its activity against *F. graminearum*. While the change in [H19A] decreased by one unit the total net charge of the peptide and increased the MIC and time–kill, the change in [H19R] maintained its charge and activity [21].

A lipid mixture was designed to imitate the plasma membrane of filamentous fungi, such as *F. graminearum*, to study the peptides’ mechanism of action on the fungal cell membrane. To do so, some modifications were made to the lipid composition used in [43]. The authors designed and used a lipid mixture composed of phosphatidylcholine (PC)/phosphatidylethanolamine (PE)/phosphatidylinositol (PI)/ergosterol (Erg) (5:4:1:2, *w*/*w*/*w*/*w*) as a representative of the yeast plasma membrane. The negatively charged lipid phosphatidylinositol was replaced by phosphatidic acid (PA) to mimic a filamentous fungal plasma membrane. PA plays a crucial role in regulating membrane curvature and membrane–cytoskeletal interactions, as a precursor of phospholipids, and might be of importance for defensin antifungal activity [44]. Moreover, PA is the molecular target of MtDef4 and HsAFP1, defensins with known antifungal activity against *F. graminearum* and *F. culmorum*, respectively [42,45,46]. The compressibility modulus at a surface pressure of 30 mN/m indicates that the mixed mixture mimics the rheological fluid character of most cellular membranes. The mixed monolayer also showed a collapse pressure of 45 ± 2 mN/m, which agrees with the collapse-pressure weighted average of pure lipid isotherms, suggesting an ideal mixing of components.

The designed model lipid membrane had a negative ζ potential value in the absence of peptides, likely related to the presence of the anionic lipid PA. Colloidal particles are metastable systems and tend to flocculate over time. Surface-charged lipid vesicles remain stable for long periods due to the electrostatic repulsion of the membrane surfaces [47]. The incorporation of defensin-derived peptides into membranes may reduce the surface charge and accelerate the vesicle aggregation process. Due to a D residue in position 21, SmAP3_α1–21_ has a Δ1.2-units-lower net charge than SmAP_α1–21_. When analyzing the LUVs model system, our results show differences between the SmAP3_α1–21_ and the SmAP_α1–21_ ζ potential neutralization powers starting from subMIC concentrations. Moreover, SmAP_α1–21_ did not induce vesicle aggregation at its MIC. Hence, we propose that a driving force for SmAP_α1–21_ membrane-targeting activity is the electrostatic interaction between negatively charged membrane lipids and positively charged amino acids.

The hydrophobicity of SmAP3_α1–21_ is observable in the hydrophaticity profile and the GRAVY value, which is lower than that of SmAP_α1–21_. This may be due to the changes in [F11S] and [G21D], amino acids with a lower hydrophobic index [48]. SmAP3_α1–21_ did not inhibit *F. graminearum* growth in the assayed conditions. In line with its modest hydrophobicity and net charge, SmAP3_α1–21_ induced a scarce increase in the ζ potential values and moderated vesicle remodeling.

Even when it presents a positive net charge (+3.8) and is the most active antifungal peptide with a MIC of 20 μM, SmAP_γ27–44_ induced small changes in the ζ potential of anionic lipid vesicles at its MIC, not supporting a classical peptide–membrane interaction as described for other AMPs [32,49]. The GRAVY value indicates that SmAP_γ27–44_ is more hydrophobic than the α-core-based peptides. When observing hydropathicity and polarity profiles, this peptide shows a hydrophobic N-terminal region and a hydrophilic C-terminal region. Its interaction with the vesicles is evident since it induces vesicle aggregation and the disruption of small lipid aggregates.

Several studies on synthetic cationic peptides provide valuable insights into peptide–membrane interactions. The hydrophilic cationic peptide nona-arginine represents an interesting model for the study of cell-penetrating peptides (CPPs). CPPs can translocate the cell membrane and reach the cellular interior [50]. The interaction of nona-arginine with different model lipid membranes has been evaluated [51]. Interestingly, anionic phospholipid vesicles vulnerable to CPP insertion and translocation show less change in ζ potential upon peptide addition than membranes resistant to peptide translocation, as was seen for vesicles challenged with SmAP_γ27–44_. This could explain this peptide localization in the cellular interior of *F. graminearum* conidia.

The high increase in the ζ potential induced by SmAP_α1–21_ may be a consequence of the surface localization of the peptide on vesicles. As seen in Fernández et al. [14] and in our confocal images with FM 4–64 dye (Figure 7), membrane perturbation and permeabilization occurred, but, as we showed when using the derivatized SmAP_γ27–44_ with *F. graminearum* conidia (Figure 8), these mechanisms seem to be distinct. On the other hand, the low ζ potential increase and the high remodeling membrane capacity of SmAP_γ27–44_ indicate a deeper location of this amphiphilic peptide in the membrane interior, inducing the destabilization of the lipid bilayer. This is supported by the high blue shift of the W emission peak when SmAP_γ27–44_ was incubated with the anionic vesicles, evidencing the translocation of the W residue to a less polar environment.

As can be seen in Table 1, each defensin-derived peptide contains a single W residue, either at the N-terminal end (SmAP_γ27–44_) or at the interior region of the peptide sequence (both α-core-derived peptides). The change in polarity of the microenvironment surrounding the indole ring occurs when the tryptophan side chain is transferred from the aqueous solution to the lipid bilayer. This transition produces a shift of W fluorescence to a smaller wavelength (blue shift) as previously reported for many lipid-interacting peptides [33], indicating a direct correlation between the insertion of the W residue into the membrane environment and their activity against *F. graminearum.* The relaxation dynamics of the W environment were assessed for the defensin-derived peptides through REES of the W fluorescence emission peak. SmAP_γ27–44_ and SmAP3_α1–21_ showed extremely high REES values in solution, evidencing a highly restricted dynamic of the dipolar W-interacting molecules. The highly restricted dynamics of the SmAP_γ27–44_ W environment suggest a fold of its structure and a strong interaction of W with the hydrophilic region of the peptide. For SmAP3_α1–21_, the W residue appears to strongly interact with the polar residues distributed uniformly on the peptide sequence. A more relaxed structure, but still dynamically restricted, is sensed by W for SmAP_α1–21_.

In the presence of anionic vesicles, the W residue of SmAP_γ27–44_ appears to restrict even more its dipolar environment. However, SmAP3_α1–21_ relaxes it to a value similar to that obtained for other membrane-interacting peptides [34,36]. Notably, the SmAP3_α1–21_ W environment appears unchanged upon vesicle addition, reinforcing the evidence of a poorer interaction with membranes. High values of REES are seldom reported in the literature, and the value obtained for SmAP_γ27–44_ is only comparable with the one obtained for the ω-loop region of the human prothrombin γ-carboxyglutamic acid domain when interacting with cationic vesicles [52]. The authors interpreted this effect as the consequence of a strong interaction of W with the heterogeneous and motion-restricted interfacial region of the phospholipid membrane.

Considering that SmAP_γ27–44_ can translocate the *F. graminearum* conidia cell membrane and shows evidence of both a deeper location in the lipid bilayer and strong interaction with the phospholipid headgroup, we propose that this peptide can act in a similar way to CPPs. Additionally, amphiphilic CPPs have been described as containing a hydrophobic motif at their N-terminal, a hydrophilic C-terminal end, an anionic domain, and a linker domain that enhances the flexibility of the hydrophobic and hydrophilic domains [50], a description that fits the SmAP_γ27–44_ structure. Molecular dynamic simulations of nona-arginine and an amphiphilic derivative containing three W residues showed that this CPP induces the formation of a hydrophilic pore whose rim, defined by the phospholipid headgroups, tightly binds to the peptide, leaving the counterions outside the membrane [53]. A similar mechanism can be pictured for SmAP_γ27–44_-membrane interaction, which can explain the experimental evidence shown here.

SmAP_α1–21_ has a strong effect on the ζ potential of anionic vesicles, vesicle aggregation capacity after reaching a concentration threshold, and moderate transfer of W to the membrane. Considering all the data presented in this work, the mode of action of SmAP_α1–21_ is different from that of SmAP_γ27–44_. The mode of action of SmAP_α1–21_ appears closer to the behavior of classical AMPs, where, after an electrostatic attraction of the cationic peptide to the anionic membrane and reaching a surface concentration threshold, the peptide induces membrane damage through the formation of membrane pores or in a detergent-like mode [32].

## 4. Materials and Methods

### 4.1. Peptide Design and Synthesis

Peptides were designed based on the α and γ-core motifs of DefSm2-D and DefSm3, which shared the γ-core sequence.

Three peptides were obtained following the method of Fernandez et al. [14]. Briefly, peptide sequences were synthesized using a Liberty Blue™ automated microwave peptide synthesizer (CEM Corp., Matthews, NC, USA) following a standard 9-fluorenyl methoxycarbonyl (Fmoc, Iris Biotech GmbH, Marktredwitz, Germany)/tert-butoxycarbonyl (tBu, Iris Biotech GmbH) protocol [54]. After cleavage from the resin Rink Amide AM (Iris Biotech GmbH), crude peptides were precipitated by adding diethyl ether. Elution was performed with a Clean-Up^®^ CEC18153 column (UCT, Bristol, PA, USA) using increasing acetonitrile concentrations in water. The collected fractions were evaporated using a Savant SPD1010 SpeedVac Concentrator (Thermo Fisher Scientific, Waltham, MA, USA). The main fractions that contained the expected peptide were determined by reversed-phase high-performance liquid chromatography (RP-HPLC) analysis performed on an XBridge™ BEH C18 column (Waters Corporation, Milford, MA, USA). Each peptide’s molecular mass was determined by MALDI-TOF mass spectrometry (Figure S1). For spectra acquisition, 1 μL of each sample was mixed with 1 μL of the α-cyano-4-hydroxycinnamic acid matrix (CHCA prepared at 10 mg/mL in 50% *v*/*v* acetonitrile and 0.1% *v*/*v* formic acid) on a microscout sample holder plate (Bruker Daltonics Inc., Billerica, MA, USA) Mass spectra were obtained with MALDI-TOF Microflex equipment (Bruker Daltonics Inc.), calibrated with an external standard for the 280–1200 *m*/*z* range, in the positive ion mode using linear detection. Reported spectra were acquired by averaging 250 laser shots (10 scans of 25 laser impacts) applied at different points taken randomly from each sample. To generate the spectra, the program mMass version 5.5.0 was used [55,56]. To detect the monoisotopic *m*/*z* signals in the spectra, the MALDI-TOF peptide’s algorithm was used with the parameters set as follows: signal/noise ratio: 3.5; relative intensity limit: 0.5%; picking height: 75%; apply baseline: enabled; apply smoothing: enabled; and apply deisotoping, disabled.

Peptides were stored as lyophilized dry powders and resuspended in milli-Q water before use. The concentration of the peptides was determined by the Beer–Lambert law: the absorbance (A) was directly proportional to the product of its molar absorptivity (ɛ), path length (b), and concentration (c). Absorbance at 280 nm was measured in an Agilent 8453 UV–visible spectrophotometer (Agilent Technologies, Santa Clara, CA, USA), and theoretical ɛ was calculated using the web tool ProtParam of ExPASy (the Expert Protein Analysis System) web service (https://web.expasy.org/protparam/, accessed on 1 October 2024).

The hydropathy and polarity profiles of the studied peptides were assessed using the ProtScale tools of the ExPASy web server (https://web.expasy.org/protscale/, accessed on 1 October 2024) [57]. These tools allowed us to compute and represent the profile generated by any amino acid scale on the protein sequence, like the polarity and hydropathy scales. The graphs were made with a window size of three amino acids, where the central amino acid of the window always weighed 100%.

Three-dimensional models were created through a neural network using ColabFold notebook AlphaFold 2 [58]. UCSF Chimera software 1.19 was used to analyze the 3D structures [59]. RMSD values of six top-ranked structures of DefSm3 were calculated against the structure of DefSm2-D using the most probable structure alignment and superposition methods (MatchMaker tools) [60]. The values shown represent the quantitative measure of the superimposed atoms [26].

### 4.2. Antifungal Activity

As previously reported, the peptides were tested for antifungal activity against the filamentous fungus *F. graminearum* through hyphal growth inhibition assays [14]. Fungal isolates were cultured under constant agitation on a carboxymethyl cellulose sporulation medium at 170 rpm and 25 °C for 5–7 days until abundant spore production occurred. Macroconidia were harvested by centrifugation at 4000 rpm and 4 °C for 15 min, then resuspended in half-strength potato dextrose broth (PDB) and adjusted to ≈10^4^ spores/mL using a hemocytometer. Aliquots (90 μL) of the spore suspension were incubated for 48 h at 25 °C in a 96-well microplate with filter-sterilized peptide solutions (10 μL) at different concentrations in water. Spore germination was assessed by measuring the optical sporulation medium density at 595 nm using the microplate reader Infinite M200 Pro (Tecan, Männedorf, Switzerland) after 0, 19, 24, 43, and 48 h of incubation. Each test was performed in triplicate. For the peptides already reported, the minimum inhibitory concentration (MIC) was tested and SmAP3_α1–21_ was tested at 50 and 100 μM. Inhibition data were analyzed by one-way ANOVA, with mean differences evaluated at *p* < 0.05 using the Tukey test. Statistical analyses were conducted using GraphPad Prism 8.0.2 software (GraphPad Software, Inc., Boston, MA, USA).

### 4.3. Model Lipid Membrane

A model membrane composed of phosphatidylcholine (PC)/phosphatidylethanolamine (PE)/phosphatidic acid (PA)/ergosterol (Erg) (5:4:1:2, *w*/*w*/*w*/*w*) (Avanti Polar Lipids, Alabaster, AL, USA) was used to mimic a fungal plasma membrane. The chosen lipid mixture was characterized by the compression isotherm of Langmuir monolayers [61]. Briefly, monomolecular lipid films were prepared by spreading appropriate aliquots of chloroform solutions of lipids onto the aqueous surface of a Teflon™ trough, using KSV NIMA minitrough equipment (Biolin Scientific AB, Gothenburg, Sweden). The surface pressure (π) was measured with a platinum plate following the Wilhelmy method, which calculates the difference between the surface tension of a bare surface and that with the monolayer. The film was compressed isometrically at a rate of 6 ± 1 Å^2^·molec^−1^·min^−1^ after solvent evaporation and relaxation, by reducing the area between two Delrin™ barriers until the collapse pressure was reached. The mean molecular area was determined by dividing the total monolayer area by the number of molecules at the interface. Surface elasticity was evaluated by calculating the compressibility modulus (Cs^−1^) using Equation (1).

(1) $${Cs}^{-1}=-A\left(\frac{\delta \pi }{\delta A}\right),$$

where π denotes the surface pressure and A the film area.

### 4.4. Liposome Preparation

Large unilamellar liposomes (LUVs) composed of PC/PE/PA/Erg 5:4:1:2 (*w*/*w*/*w*/*w*) were used as models of fungal plasma membranes. LUV formation was performed as previously reported [62]. Multilamellar vesicles were prepared by creating a uniform lipid film on the wall of a glass test tube from 1 mM lipids dissolved in chloroform/methanol (2:1) through solvent evaporation under a nitrogen stream. Any remaining solvent traces were removed by placing the tube in a high-vacuum chamber for 1 h. The lipids were then hydrated with 5 mM HEPES buffer at pH 7.0 while applying vigorous mixing and nine freezing–thawing cycles (at 0 °C and 40 °C, respectively). LUVs with an average diameter of 100 nm were prepared by extruding the multilamellar vesicles 31 times through polycarbonate filters of a 100 nm pore size at room temperature.

### 4.5. Particle Analysis

The particle-size distribution of LUVs at different peptide concentrations was analyzed by dynamic light scattering (DLS) with a Nicomp™ 380 Submicron Particle Sizer (Santa Barbara, CA, USA) using a 530 nm laser beam, and analyzed at a 90° angle via the correlation function adjustment. The hydrodynamic diameter of the particles was calculated using the Stokes–Einstein equation. The ζ potential measurements were performed using the Zetasizer SZ-100-Z system (Horiba Ltd., Kioto, Japan), equipped with a solid-state semiconductor laser (532 nm, 10 mW) and using the laser Doppler velocimetry technique [61]. LUV suspensions were analyzed at a 0.1 mM lipid concentration and 0, 15, 20, 30, 40, and 50 µM peptide concentrations. The samples were evaluated by quintuple repetitions per condition in two independent assays.

### 4.6. Red Edge Excitation Shift (REES) Experiments

REES experiments assessed the environment around the W residue [36]. W fluorescence emission spectra were collected with a Spectrofluorometer Fluoromax-P (Horiba Ltd.), using a quartz cell with a 0.1 cm path length, thermostated at 25 °C. Emission spectra were recorded 1 h after sample preparation. Excitation and emission slits of 2.0 nm along with a bandpass filter were used. Scattering corrections were made by subtracting spectra obtained at each vesicle concentration, which served as the blank. For each sample, fluorescence emission spectra were collected with a sweep of excitation wavelengths from 280 to 310 nm, monitoring emission from 300 to 400 nm with a variation of 0.5 nm. Measurements were made at a 50 μM peptide concentration and 1 mM lipid concentration. Three measurements were averaged to obtain each spectrum.

### 4.7. SmAP_γ27–44_ Derivatization

Once peptide synthesis was completed and before the cleavage and deprotection of side groups, one batch of the peptide SmAP_γ27–44_ was derivatized with fluorescein 5(6)-carboxyfluorescein (Novabiochem^®^, Merck KGaA, Darmstadt, Germany). The probe was incorporated as described in Fernandez et al. [21] after the deprotection of the last amino acid coupled to the peptidyl resin. After probe incorporation, side chains were deprotected, and the peptide was cleaved from the polymeric support. The crude labeled peptides were precipitated with diethyl ether, washed with ethanol, dried, dissolved in ultrapure water, frozen, and lyophilized. Then, the labeled peptide was purified following the same procedure as for unlabeled peptides. Labeled SmAP_γ27–44_ was obtained at 30% acetonitrile, while a residual fraction of unlabeled peptide was eluted at 10% acetonitrile.

### 4.8. Analysis by Confocal Laser Scanning Microscopy (CLSM) and Subcellular Localization

CLSM was performed to monitor the potential internalization and subcellular localization of the fluorescently labeled SmAP_γ27–44_ in *F. graminearum* conidia using a Leica TCS SP5 CLSM (Leica Microsystems GmbH, Wetzlar, Germany) in a dark room at 20 °C. Before testing, the MIC of the labeled peptide was checked to evaluate if it coincided with that of the unlabeled peptide. Conidia were treated with fluorescein peptide at its MIC and incubated for 45 min at 25 °C in the dark, before mounting on a microscope for imaging. Fluorescein peptide was excited at 488 nm using an argon ion laser, and fluorescence was detected in the range of 506–566 nm. Bright-field images were captured with a transmitted light detector. Images were then processed with Leica Confocal Software (LCS) Lite v. 2.61.15.

Conidia were exposed to unlabeled SmAP_α1–21_ and SmAP_γ27–44_ at their MIC for at least 45 and 30 min (depending on the time–kill of each peptide). After treatment, 3 µM FM™ 4–64 dye (Invitrogen™, ThermoFisher Scientific Inc.) was added and the preparation was immediately assembled on the slide for visualization in the CLSM Leica TCS SP5. FM™ 4–64 dye was excited at 488 nm using an argon ion laser and detected in the range of 560–620 nm. Water and 80 µM cetrimide were the negative and positive controls, respectively.

## 5. Conclusions

The interaction of defensin-derived peptides with model lipid membranes and the interaction with cells was studied to deepen the understanding of the mechanism of action of these peptides and their relationship with their chemical and physical features. A notable direct relationship was found between the hydropathicity and membrane-penetrating capacity of the peptides with their antifungal activity against *F. graminearum*. Interestingly, while a cell-penetrating peptide-like effect was pictured for the peptide derived from the γ-core of defensin, a classical AMP action was observed for the α-core derived peptide. Both peptides perturb the plasma membrane and enter the fungal cell producing vesiculation, but SmAP_γ27–44_ localizes to specific subcellular regions.

## Figures and Tables

**Table 1:** Main properties of the studied defensin-derived peptides. The peptide sequences show the presence and position of the fluorescent amino acid residue tryptophan (W, yellow); the cationic amino acids arginine, histidine, and lysine (R, H, and K in cyan); the anionic amino acids aspartic acid and glutamic acid (D, E, in magenta); the S-S bond-forming amino acid cysteine (C, red); and the structure-breaking amino acid proline (P, gray).

Peptide Name	Sequence	Molecular Weight ^1^ (Da)	pI ^2^	Net Charge ^3^	GRAVY Value ^4^	MIC (μM) ^5^	Time to Kill (min)
SmAP_γ27–44_	WEGAVHGACHVRNGKHMC	1991.5	8.1 *	3.8 *	−0.938	20 *	30 *
SmAP_α1–21_	KLCEKPSKTWFGNCGNPRHCG	2361.6	9.0 *	4 *	−1.076	32 *	60–180 *
SmAP3_α1–21_	KLCEKPSKTWSGNCGNTHHCD	2348.1	8.0	2.8	−1.290	>100	nd

* Data extracted from Ref. [14]. ^1^ MALDI-TOF spectra of purified peptides are shown in Figure S1 (Supplementary Materials). ^2^ Theoretical isoelectric point (pI) was calculated using the ExPASy tool Compute pI/MW. ^3^ Net charge was calculated at pH 5.5 (pH of half strength PDB [potato dextrose broth] in which the antifungal test was performed). ^4^ The grand average of hydropathy (GRAVY) value for a peptide or protein is calculated as the sum of hydropathy values of all the amino acids, divided by the number of residues in the sequence. It was calculated using the ExPASy tool ProtParam. ^5^ Minimum inhibitory concentration (MIC) is the minimal peptide concentration that completely inhibits *F. graminearum* growth. nd: not determined.

**Table 2:** W fluorescence red edge excitation shift (REES) and blue shift values for each peptide in the absence and presence of LUVs (1 mM).

Peptide	Emission Peak Shift (Ex 280 nm)	REES Peptide (nm)	REES Peptide + LUVs (nm)
SmAP_γ27–44_	17 ± 6	26 ± 5	38 ± 2
SmAP_α1–21_	14 ± 3	17 ± 2	11 ± 3
SmAP3_α1–21_	7 ± 2	28 ± 4	28 ± 3

## Data Availability

The original contributions presented in the study are included in the article/Supplementary Materials, further inquiries can be directed to the corresponding author/s.

## Supplementary Materials

The following supporting information can be downloaded at: https://www.mdpi.com/article/10.3390/antibiotics14050430/s1, Figure S1: MALDI-TOF spectra of purified peptides; Figure S2: Isotherms for pure lipids and lipid mixture on a subphase containing 145 mM NaCl at 25 °C.
