# Supplementary material for: Antifungal Peptides SmAPα1–21 and SmAPγ27–44 Designed from Different Loops of DefSm2-D Have Distinct Modes of Action
Source: Antibiotics (Basel). 2025 Apr 24;14(5):430. doi: 10.3390/antibiotics14050430 (PMC12108439; doi:10.3390/antibiotics14050430)
Supplement: Supplementary file 1 [file antibiotics-14-00430-s001.zip › antibiotics-3266276-supplementary.pdf]

Supplementary Material

Peptide-membrane interaction of antifungal peptides derived from *Silybum marianum* defensins

Micaela Iturralde; Juan P. Bracho; Jessica A. Valdivia-Pérez; Fanny Guzmán; Ismael Malbrán; Sabina Maté; María Laura Fanani; and Sandra Vairo Cavalli

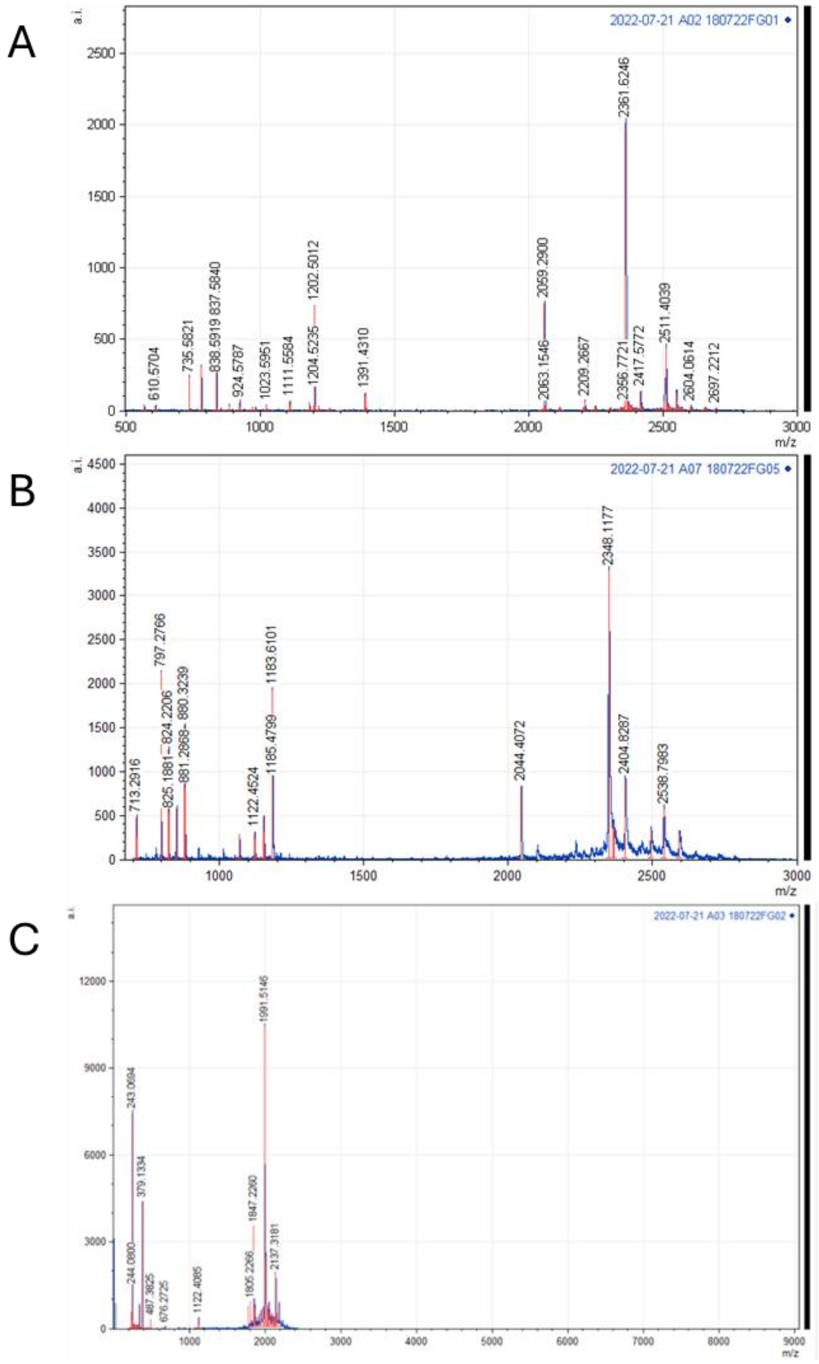

Figure S1. MALDI-TOF spectra of purified peptides. SmAP$_{\alpha1-21}$ (A), SmAP3$_{\alpha1-21}$ (B), and SmAP$_{\gamma27-44}$ (C). The highest peak is the one corresponding to the mass of the peptide.

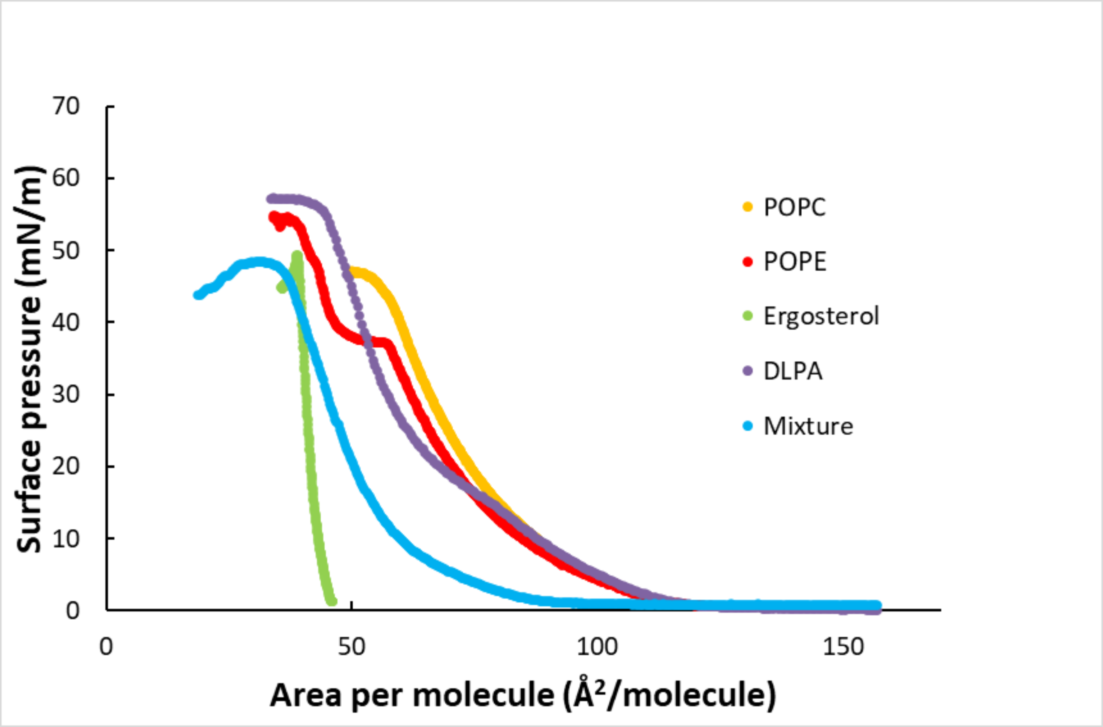

Figure S2. Isotherms for pure lipids and lipid mixture on a subphase containing 145 mM NaCl at 25°C. POPC (orange), POPE (red), ergosterol (green), DMPA (violet), and lipid mixture (cyan).
